# Analysis of the COVID-19 pandemic: lessons towards a more effective response to public health emergencies

**DOI:** 10.1186/s12992-022-00805-9

**Published:** 2022-02-04

**Authors:** Yibeltal Assefa, Charles F. Gilks, Simon Reid, Remco van de Pas, Dereje Gedle Gete, Wim Van Damme

**Affiliations:** 1 School of Public Health, the University of Queensland, Brisbane, Australia; 2 Institute of Tropical Medicine, Antwerp, Belgium

**Keywords:** Pandemics, Epidemics, COVID-19, Heterogeneity, Governance, Equity

## Abstract

**Background:**

The pandemic of Coronavirus Disease 2019 (COVID-19) is a timely reminder of the nature and impact of Public Health Emergencies of International Concern. As of 12 January 2022, there were over 314 million cases and over 5.5 million deaths notified since the start of the pandemic. The COVID-19 pandemic takes variable shapes and forms, in terms of cases and deaths, in different regions and countries of the world. The objective of this study is to analyse the variable expression of COVID-19 pandemic so that lessons can be learned towards an effective public health emergency response.

**Methods:**

We conducted a mixed-methods study to understand the heterogeneity of cases and deaths due to the COVID-19 pandemic. Correlation analysis and scatter plot were employed for the quantitative data. We used Spearman’s correlation analysis to determine relationship strength between cases and deaths and socio-economic and health systems. We organized qualitative information from the literature and conducted a thematic analysis to recognize patterns of cases and deaths and explain the findings from the quantitative data.

**Results:**

We have found that regions and countries with high human development index have higher cases and deaths per million population due to COVID-19. This is due to international connectedness and mobility of their population related to trade and tourism, and their vulnerability related to older populations and higher rates of non-communicable diseases. We have also identified that the burden of the pandemic is also variable among high- and middle-income countries due to differences in the governance of the pandemic, fragmentation of health systems, and socio-economic inequities.

**Conclusion:**

The COVID-19 pandemic demonstrates that every country remains vulnerable to public health emergencies. The aspiration towards a healthier and safer society requires that countries develop and implement a coherent and context-specific national strategy, improve governance of public health emergencies, build the capacity of their (public) health systems, minimize fragmentation, and tackle upstream structural issues, including socio-economic inequities. This is possible through a primary health care approach, which ensures provision of universal and equitable promotive, preventive and curative services, through whole-of-government and whole-of-society approaches.

## Background

The pandemic of Coronavirus Disease 2019 (COVID-19) is a timely reminder of the nature and impact of emerging infectious diseases that become Public Health Emergency of International Concern (PHEIC) [1]. The COVID-19 pandemic takes variable shapes and forms in how it affects communities in different regions and countries [2, 3]. As of 12 January, 2022, there were over 314 million cases and over 5.5 million deaths notified around the globe since the start of the pandemic. The number of cases per million population ranged from 7410 in Africa to 131,730 in Europe while the number of deaths per million population ranged from 110 in Oceania to 2740 in South America. Case-fatality rates (CFRs) ranged from 0.3% in Oceania to 2.9% in South America [4, 5]. Regions and countries with high human development index (HDI), which is a composite index of life expectancy, education, and per capita income indicators [6], are affected by COVID-19 more than regions with low HDI. North America and Europe together account for 55 and 51% of cases and deaths, respectively. Regions with high HDI are affected by COVID-19 despite their high universal health coverage index (UHCI) and Global Health Security index (GHSI) [7].

This seems to be a paradox (against the established knowledge that countries with weak (public) health systems capacity will have worse health outcomes) in that the countries with higher UHCI and GHSI have experienced higher burdens of COVID-19 [7]. The paradox can partially be explained by variations in testing algorithms, capacity for testing, and reporting across different countries. Countries with high HDI have health systems with a high testing capacity; the average testing rate per million population is less than 32, 000 in Africa and 160,000 in Asia while it is more than 800, 000 in HICs (Europe and North America). This enables HICs to identify more confirmed cases that will ostensibly increase the number of reported cases [3]. Nevertheless, these are insufficient to explain the stark differences between countries with high HDI and those with low HDI. Many countries with high HDI have a high testing rate and a higher proportion of symptomatic and severe cases, which are also associated with higher deaths and CFRs [7]. On the other hand, there are countries with high HDI that sustain a lower level of the epidemic than others with a similar high HDI. It is, therefore, vital to analyse the heterogeneity of the COVID-19 pandemic and explain why some countries with high HDI, UHCI and GHSI have the highest burden of COVID-19 while others are able to suppress their epidemics and mitigate its impacts.

The objective of this study was to analyse the COVID-19 pandemic and understand its variable expression with the intention to learn lessons for an effective and sustainable response to public health emergencies. We hypothesised that high levels of HDI, UHCI and GHSI are essential but not sufficient to prevent and control COVID-19.

## Methods

We conducted an explanatory mixed-methods study to understand and explain the heterogeneity of the pandemic around the world. The study integrated quantitative and qualitative secondary data. The following steps were included in the research process: (i) collecting and analysing quantitative epidemiological data, (ii) conducting literature review of qualitative secondary data and (iii) evaluating countries’ pandemic responses to explain the variability in the COVID-19 epidemiological outcomes. The study then illuminated specific factors that were vital towards an effective and sustainable epidemic response.

We used the publicly available secondary data sources from Johns Hopkins University (https://coronavirus.jhu.edu/data/new-cases) for COVID-19 and UNDP 2020 HDI report (http://hdr.undp.org/en/2019-report) for HDI, demographic and epidemiologic variables. These are open data sources which are regularly updated and utilized by researchers, policy makers and funders. We performed a correlation analysis of the COVID-19 pandemic. We determined the association between COVID-19 cases, severity, deaths and CFRs at the 0.01 and 0.05 levels (2-tailed). We used Spearman’s correlation analysis, as there is no normal distribution of the variables [8].

The UHCI is calculated as the geometric mean of the coverage of essential services based on 17 tracer indicators from: (1) reproductive, maternal, newborn and child health; (2) infectious diseases; (3) non-communicable diseases; and, (4) service capacity and access and health security [9]. The GHSI is a composite measure to assess a country’s capability to prevent, detect, and respond to epidemics and pandemics [10].

We then conducted a document review to explain the epidemic patterns in different countries. Secondary data was obtained from peer-reviewed journals, reputable online news outlets, government reports and publications by public health-related associations, such as the WHO. To explain the variability of COVID-19 across countries, a list of 14 indicators was established to systematically assess country’s preparedness, actual pandemic response, and overall socioeconomic and demographic profile in the context of COVID-19. The indicators used in this study include: 1) Universal Health Coverage Index, 2) public health capacity, 3) Global Health Security Index, 4) International Health Regulation, 5) leadership, governance and coordination of response, 6) community mobilization and engagement, 7) communication, 8) testing, quarantines and social distancing, 9) medical services at primary health care facilities and hospitals, 10) multisectoral actions, 11) social protection services, 12) absolute and relative poverty status, 13) demography, and 14) burden of communicable and non-communicable diseases. These indicators are based on our previous studies and recommendation from the World Health Organization [3, 4]. We conducted thematic analysis and synthesis to identify the factors that may explain the heterogeneity of the pandemic.

## Results

### Heterogeneity of COVID-19 cases and deaths around the world: what can explain it?

**Fig. 1:**
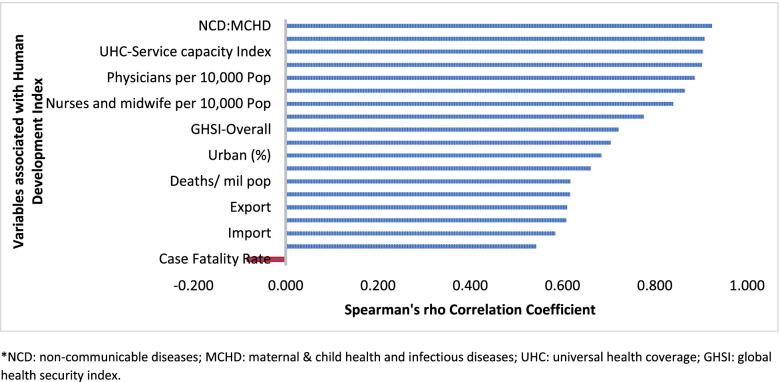
Human development index and its correlates associated with COVID-19 in 189 countries*

Table 1 indicates that the pandemic of COVID-19 is heterogeneous around regions of the world. Figure 1 also shows that there is a strong and significant correlation between HDI and globalisation (with an increase in trade and tourism as proxy indicators) and a corresponding strong and significant correlation with COVID-19 burden.

**Table 1:** COVID-19 cases, deaths and case-fatality rates in six regions of the world

Regions	Cases per mil pop	Deaths per mil pop	Total cases (%)	Total deaths (%)	Tests per mil pop	Case-fatality rate^a^
**Europe**	131,730	2082	31%	28%	2,379,478	1.6%
**North America**	125,015	2121	24%	23%	1,587,217	1.7%
**South America**	95,342	2740	13%	22%	436,876	2.9%
**Oceania**	32,673	110	0.4%	0.1%	149,348	0.3%
**Asia**	18,813	272	28%	23%	372,822	1.4%
**Africa**	7410	167	3%	4%	64,564	2.3%
**World**	**40,295**	**708**	**100%**	**100%**	**519,312**	**1.8%**

Source: worldometer- COVID-19 coronavirus pandemic: https://www.worldometers.info/coronavirus/

^a^Case-fatality rate is calculated as a percentage of reported deaths out of reported cases of COVID-19

Globalisation and pandemics interact in various ways, including through international trade and mobility, which can lead to multiple waves of infections [11]. In at least the first waves of the pandemic, countries with high import and export of consumer goods, food products and tourism have high number of cases, severe cases, deaths and CFRs. Countries with high HDI are at a higher risk of importing (and exporting) COVID-19 due to high mobility linked to trade and tourism, which are drivers of the economy. These may have led to multiple introductions of COVID-19 into these countries before border closures.

The COVID-19 pandemic was first identified in China, which is central to the global network of trade, from where it spread to all parts of the world, especially those countries with strong links with China [12]. The epidemic then spread to Europe. There is very strong regional dimension to manufacturing and trading, which could be facilitate the spread of the virus. China is the heart of ‘Factory Asia’; Italy is in the heart of ‘Factory Europe’; the United States is the heart of ‘Factory North America’; and Brazil is the heart of ‘Factory Latin America’ [13]. These are the countries most affected by COVID-19 during the first wave of the pandemic [2, 3, 14].

It is also important to note that two-third of the countries currently reporting more than a million cases are middle-income countries (MICs), which are not only major emerging market economies but also regional political powers, including the BRICS countries (Brazil, Russia, India and South Africa) [3, 15]. These countries participate in the global economy, with business travellers and tourists. They also have good domestic transportation networks that facilitate the internal spread of the virus. The strategies that helped these countries to become emerging markets also put them at greater risk for importing and spreading COVID-19 due to their connectivity to the rest of the world.

In addition, countries with high HDI may be more significantly impacted by COVID-19 due to the higher proportion of the elderly and higher rates of non-communicable diseases. Figure 1 shows that there is a strong and significant correlation between HDI and demographic transition (high proportion of old-age population) and epidemiologic transition (high proportion of the population with non-communicable diseases). Countries with a higher proportion of people older than 65 years and NCDs (compared to communicable diseases) have higher burden of COVID-19 [16–20]. Evidence has consistently shown a higher risk of severe COVID-19 in older individuals and those with underlying health conditions [21–25]. CFR is age-dependent; it is highest in persons aged ≥85 years (10 to 27%), followed by those among persons aged 65–84 years (3 to 11%), and those among persons aged 55-64 years (1 to 3%) [26].

On the other hand, regions and countries with low HDI have, to date, experienced less severe epidemics. For instance, as of January 12, 2022, the African region has recorded about 10.3 million cases and 233,000 deaths– far lower than other regions of the world (Table 1) [27]. These might be due to lower testing rates in Africa, where only 6.5% of the population has been tested for the virus [14, 28], and a greater proportion of infections may remain asymptomatic [29]. Indeed, the results from sero-surveys in Africa show that more than 80% of people infected with the virus were asymptomatic compared to an estimated 40-50% asymptomatic infections in HICs [30, 31]. Moreover, there is a weak vital registration system in the region indicating that reports might be underestimating and underreporting the disease burden [32]. However, does this fully explain the differences observed between Africa and Europe or the Americas?

Other possible factors that may explain the lower rates of cases and deaths in Africa include: (1) Africa is less internationally connected than other regions; (2) the imposition of early strict lockdowns in many African countries, at a time when case numbers were relatively small, limited the number of imported cases further [2, 33, 34]; (3) relatively poor road network has also limited the transmission of the virus to and in rural areas [35]; (4) a significant proportion of the population resides in rural areas while those in urban areas spend a lot of their time mostly outdoors; (5) only about 3% of Africans are over the age of 65 (so only a small proportion are at risk of severe COVID-19) [36]; (6) lower prevalence of NCDs, as disease burden in Africa comes from infectious causes, including coronaviruses, which may also have cross-immunity that may reduce the risk of developing symptomatic cases [37]; and (7) relative high temperature (a major source of vitamin D which influences COVID-19 infection and mortality) in the region may limit the spread of the virus [38, 39]. We argue that a combination of all these factors might explain the lower COVID-19 burden in Africa.

The early and timely efforts by African leaders should not be underestimated. The African Union, African CDC, and WHO convened an emergency meeting of all African ministers of health to establish an African taskforce to develop and implement a coordinated continent-wide strategy focusing on: laboratory; surveillance; infection prevention and control; clinical treatment of people with severe COVID-19; risk communication; and supply chain management [40]. In April 2021, African Union and Africa CDC launched the Partnerships for African Vaccine Manufacturing (PAVM), framework to expanding Africa’s vaccine manufacturing capacity for health security [41].

### Heterogeneity of the pandemic among countries with high HDI: what can explain it?

**Fig. 2:**
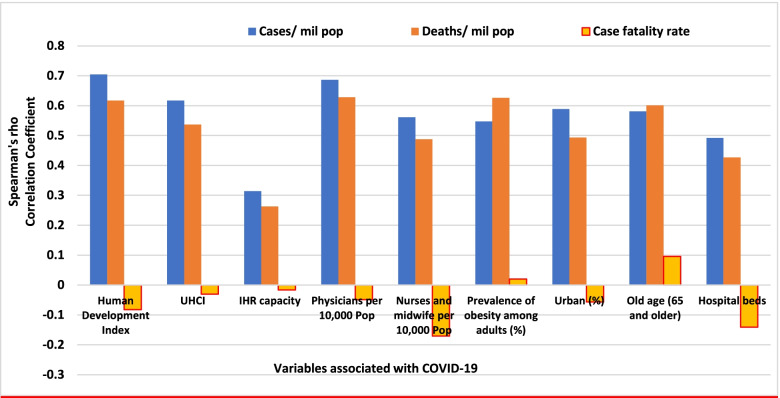
Scatter plot of COVID-19 cases per million population in countries with high human development index (> 0.70)

**Fig. 3:**
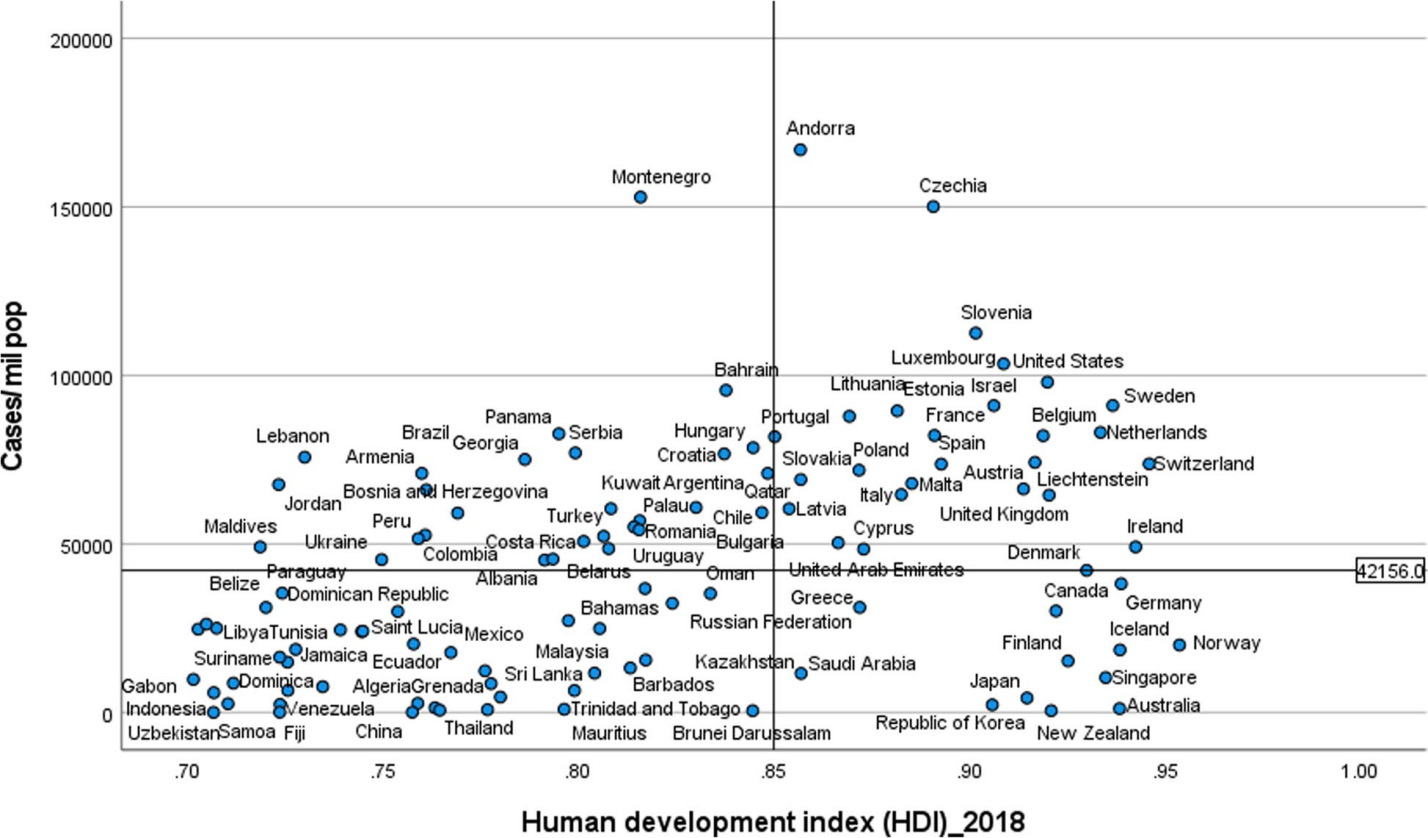
Scatter plot of COVID-19 deaths per million population in countries with high human development index (> 0.70)

Figures 2 and 3 illustrate the variability of cases and deaths due to the COVID-19 pandemic across high-income countries (HICs). Contrary to the overall positive correlation between high HDI and cases, deaths and fatality rates due to COVID-19, there are outlier HICs, which have been able to control the epidemic. Several HICs, such as New Zealand, Australia, South Korea, Japan, Denmark, Iceland, and Norway, managed to contain their epidemics (Figs. 2 and 3) [15, 42, 43]. It is important to note that most of these countries (especially the island states) have far less cross-border mobility than other HICs.

HICs that have been successful at controlling their epidemics have similar characteristics, which are related to governance of the response [44], synergy between UHC and GHS, and existing relative socio-economic equity in the country. Governance and leadership is a crucial factor to explain the heterogeneity of the epidemic among countries with high HDI [45]. There has been substantial variation in the nature and timing of the public health responses implemented [46]. Adaptable and agile governments seem better able to respond to their epidemics [47, 48]. Countries that have fared the best are the ones with good governance and public support [49]. Countries with an absence of coherent leadership and social trust have worse outcomes than countries with collective action, whether in a democracy or autocracy, and rapid mobilisation of resources [50]. The erosion of trust in the United States government has hurt the country’s ability to respond to the COVID-19 crisis [51, 52]. The editors of the New England Journal of Medicine argued that the COVID-19 crisis has produced a test of leadership; but, the leaders in the United States had failed that test [47].

COVID-19 has exposed the fragility of health systems, not only in the public health and primary care, but also in acute and long-term care systems [49]. Fragmentation of health systems, defined here to mean inadequate synergy and/ or integration between GHS and UHC, is typical of countries most affected by the COVID-19 pandemic. Even though GHS and UHC agendas are convergent and interdependent, they tend to have different policies and practices [53]. The United States has the highest index for GHS preparedness; however, it has reported the world’s highest number of COVID-19 cases and deaths due to its greatly fragmented health system [54, 55]. Countries with health systems and policies that are able to integrate International Health Regulations (IHR) core capacities with primary health care (PHC) services have been effective at mitigating the effects of COVID-19 [50, 53]. Australia has been able to control its COVID-19 epidemic through a comprehensive primary care response, including protection of vulnerable people, provision of treatment and support services to affected people, continuity of regular healthcare services, protection and support of PHC workers and primary care services, and provision of mental health services to the community and the primary healthcare workforce [56]. Strict implementation of public health and social intervention together with UHC systems have ensured swift control of the epidemics in Singapore, South Korea, and Thailand [57].

The heterogeneity of cases and deaths, due to COVID-19, is also explained by differences in levels of socio-economic inequalities, which increase susceptibility to acquiring the infection and disease progression as well as worsening of health outcomes [58]. COVID-19 has been a stress test for public services and social protection systems. There is a higher burden of COVID-19 in Black, Asian and Minority Ethnic individuals due to socio-economic inequities in HICs [59, 60]. Poor people are more likely to live in overcrowded accommodation, are more likely to have unstable work conditions and incomes, have comorbidities associated with poverty and precarious living conditions, and reduced access to health care [59].

The epidemiology of COVID-19 is also variable across MICs, with HDI between 0.70 and 0.85, around the world. Overall, the epidemic in MICs is exacerbated by the rapid demographic and epidemiologic transitions as well as high prevalence of obesity. While India and Brazil witnessed rapidly increasing rates of cases and deaths, China, Thailand, Vietnam have experienced a relatively lower disease burden [15]. This heterogeneity may be attributed to a number of factors, including governance, communication and service delivery. Thailand, China and Vietnam have implemented a national harmonized strategic response with decentralized implementation through provincial and district authorities [61]. Thailand increased its testing capacity from two to over 200 certified facilities that could process between 10,000 to 100,000 tests per day; moreover, over a million village health volunteers in Thailand supported primary health services [62, 63]. China’s swift and decisive actions enabled the country to contain its epidemic though there was an initial delay in detecting the disease. China has been able to contain its epidemic through community-based measures, very high public cooperation and social mobilization, strategic lockdown and isolation, multi-sector action [64]. Overall, multi-level governance (effective and decisive leadership and accountability) of the response, together with coordination of public health and socio-economic services, and high levels of citizen adherence to personal protection, have enabled these countries to successfully contain their epidemics [61, 65, 66].

On the other hand, the Brazilian leadership was denounced for its failure to establish a national surveillance network early in the pandemic. In March 2020, the health minister was reported to have stated that mass testing was a waste of public funding, and to have advised against it [67]. This was considered as a sign of a collapse of public health leadership, characterized by ignorance, neoliberal authoritarianism [68]. There were also gaps in the public health capacity in different municipalities, which varied greatly, with a considerable number of Brazilian regions receiving less funding from the federal government due to political tension [69]. The epidemic has a disproportionate adverse burden on states and municipalities with high socio-economic vulnerability, exacerbated by the deep social and economic inequalities in Brazil [70].

India is another middle-income country with a high burden of COVID-19. It was one of the countries to institute strict measures in the early phase of the pandemic [71, 72]. However, the government eased restrictions after the claim that India had beaten the pandemic, which lead to a rapid increase in disease incidence. Indeed, on 12 January 2022, India reported 36 million cumulative cases and almost 485,000 total deaths [15]. The second wave of the epidemic in India exposed weaknesses in governance and inadequacies in the country’s health and other social systems [73]. The nature of the Indian federation, which is highly centripetal, has prevented state and local governments from tailoring a policy response to suit local needs. A centralized one-size-fits-all strategy has been imposed despite high variations in resources, health systems capacity, and COVID-19 epidemics across states [74]. There were also loose social distancing and mask wearing, mass political rallies and religious events [75]. Rapid community transmission driven by high population density and multigenerational households has been a feature of the current wave in India [76]. In addition, several new variants of the virus, including the UK (B.1.1.7), the South Africa (20H/501Y or B.1.351), and Brazil (P.1), alongside a newly identified Indian variant (B.1.617), are circulating in India and have been implicated as factors in the second wave of the pandemic [75, 76].

### Heterogeneity of case-fatality rates around the world: what can explain it?

**Fig. 4:**
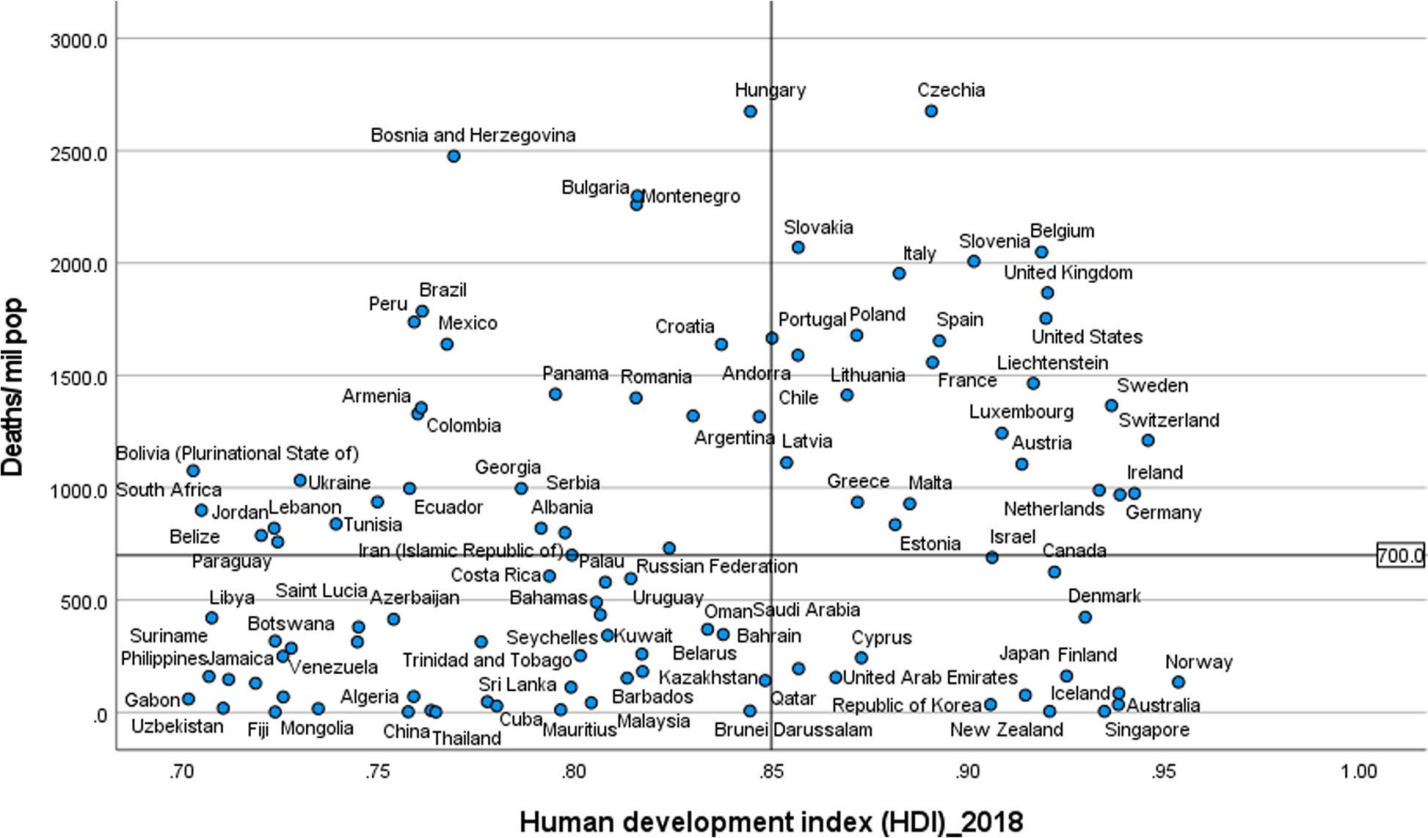
Correlates of COVID-19 cases, deaths and case-fatality rates in 189 countries

The pandemic is characterized by variable CFRs across regions and countries that are negatively associated with HDI (Fig. 1). The results presented in Fig. 4 show that the proportion of elderly population and rate of obesity are important factors which are positively associated with CFR. On the other hand, UHC, IHR capacity and other indicators of health systems capacity (health workforce density and hospital beds) are negatively associated with the CFR (Figs. 1 and 4).

The evidence from several research indicates that heterogeneity can be explained by several factors, including differences in age-pyramid, socio-economic status, access to health services, or rates of undiagnosed infections. Differences in age-pyramid may explain some of the observed variation in epidemic severity and CFR between countries [77]. CFRs across countries look similar when taking age into account [78]. The elderly and other vulnerable populations in Africa and Asia are at a similar risk as populations in Europe and Americas [79]. Data from European countries suggest that as high as 57% of all deaths have happened in care homes and many deaths in the US have also occurred in nursing homes. On the other hand, in countries such as Mexico and India, individuals < 65 years contributed the majority of deaths [80].

Nevertheless, CFR also depends on the quality of hospital care, which can be used to judge the health system capacity, including the availability of healthcare workers, resources, and facilities, which affects outcomes [81]. The CFR can increase if there is a surge of infected patients, which adds to the strain on the health system [82]. COVID-19 fatality rates are affected by numerous health systems factors, including bed capacity, existence and capacity of intensive care unit (ICU), and critical care resources (such as oxygen and dexamethasone) in a hospital. Regions and countries with high HDI have a greater number of acute care facilities, ICU, and hospital bed capacities compared to lower HDI regions and countries [83]. Differences in health systems capacity could explain why North America and Europe, which have experienced much greater number of cases and deaths per million population, reported lower CFRs than the Southern American and the African regions, partly also due to limited testing capacity in these regions (Table 1) [84–86]. The higher CFR in Southern America can be explained by the relatively lower health systems surge capacity that could not adequately respond to the huge demand for health services [69, 86]. The COVID-19 pandemic has highlighted existing health systems’ weaknesses, which are not able to effectively prepare for and respond to PHEs [87]. The high CFRs in the region are also exacerbated by the high social inequalities [69].

On the other hand, countries in Asia recorded lower CFRs (~ 1.4%) despite sharing many common risk factors (including overcrowding and poverty, weak health system capacity etc) with Africa. The Asian region shares many similar protective factors to the African region. They have been able to minimize their CFR by suppressing the transmission of the virus and flattening the epidemic curve of COVID-19 cases and deaths. Nevertheless, the epidemic in India is likely to be different because it has exceeded the health system capacity to respond and provide basic medical care and medical supplies such as oxygen [88]. Overall, many Asian countries were able to withstand the transmission of the virus and its effect due to swift action by governments in the early days of the pandemic despite the frequency of travel between China and neighbouring countries such as Hong Kong, Taiwan and Singapore [89]. This has helped them to contain the pandemic to ensure case numbers remain within their health systems capacity. These countries have benefited from their experience in the past in the prevention and control of epidemics [90].

There are a number of issues with the use of the CFR to compare the management of the pandemic between countries and regions [91], as it does not depict the true picture of the mortality burden of the pandemic. A major challenge with accurate calculation of the CFR is the denominator on number of identified cases, as asymptomatic infections and patients with mild symptoms are frequently left untested, and therefore omitted from CFR calculations. Testing might not be widely available, and proactive contact tracing and containment might not be employed, resulting in a smaller denominator, and skewing to a higher CFR [82]. It is, therefore, far more relevant to estimate infection fatality rate (IFR), the proportion of all infected individuals who have died due to the infection [91], which is central to understanding the public health impact of the pandemic and the required policies for its prevention and control [92].

Estimates of prevalence based on sero-surveys, which includes asymptomatic and mildly symptomatic infections, can be used to estimate IFR [93]. In a systematic review of 17 studies, seroprevalence rates ranged from 0.22% in Brazil to 53% in Argentina [94]. The review also identified that the seroprevalence estimate was higher than the cumulative reported case incidence, by a factor between 1.5 times in Germany to 717 times in Iran, in all but two studies (0.56 times in Brazil and 0.88 times in Denmark) [94, 95]. The difference between seroprevalence and cumulative reported cases might be due to asymptomatic cases, atypical or pauci-symptomatic cases, or the lack of access to and uptake of testing [94]. There is only a modest gap between the estimated number of infections from seroprevalence surveys and the cumulative reported cases in regions with relatively thorough symptom-based testing. Much of the gap between reported cases and seroprevalence is likely to be due to undiagnosed symptomatic or asymptomatic infections [94].

### Collateral effects of the COVID-19 pandemic

It is important to note that the pandemic has significant collateral effects on the provision of essential health services, in addition to the direct health effects [96]. Disruptions in the provision of essential health services, due to COVID-19, were reported by nearly all countries, though it is more so in lower-income than higher-income countries [97, 98]. The biggest impact reported is on provision of day-to-day primary care to prevent and manage some of the most common health problems [99].

The causes of disruptions in service delivery were a mix of demand and supply factors [100]. Countries reported that just over one-third of services were disrupted due to health workforce-related reasons (the most common causes of service disruptions), supply chains, community mistrust and fears of becoming infected, and financial challenge s[101]. Cognizant of the disruptive effects of the pandemic, countries have reorganized their health system.

Countries with better response to COVID-19 have mobilized, trained and reallocated their health workforce in addition to hiring new staff, using volunteers and medical trainees and mobilizing retirees [102]. Several strategies have also been implemented to mitigate disruptions in service delivery and utilization, including: triaging to identify the most urgent patient needs, and postponing elective medical procedures; switching to alternative models of care, such as providing more home-based care and telemedicine [101].

## Discussion

This study identifies that the COVID-19 pandemic, in terms f cases and deaths, is heterogeneous around the world. This variability is explained by differences in vulnerability, preparedness, and response. It confirms that a high level of HDI, UHCI and GHSI are essential but not sufficient to control epidemics [103]. An effective response to public health emergencies requires a joint and reinforcing implementation of UHC, health emergency and disease control priorities [104, 105], as well as good governance and social protection systems [106]. Important lessons have been learned to cope better with the COVID-19 pandemic and future emerging or re-emerging pandemics. Countries should strengthen health systems, minimize fragmentation of public health, primary care and secondary care, and improve coordination with other sectors. The pandemic has exposed the health effects of longstanding social inequities, which should be addressed through policies and actions to tackle vulnerability in living and working conditions [106].

The shift in the pandemic epicentre from high-income to MICs was observed in the second global wave of the pandemic. This is due to in part to the large-scale provision of vaccines in HICs [15] as well as the limitations in the response in LMICs, including inadequate testing, quarantine and isolation, contact tracing, and social distancing. The second wave of the pandemic in low- and middle-income countries spread more rapidly than the first wave and affected younger and healthier populations due to factors, including poor government decision making, citizen behaviour, and the emergence of highly transmissible SARS-CoV-2 variants [107]. It has become catastrophic in some MICs to prematurely relax key public health measures, such as mask wearing, physical distancing, and hand hygiene [108].

There is consensus that global vaccination is essential to ending the pandemic. Universal and equitable vaccine delivery, implemented with high volume, speed and quality, is vital for an effective and sustainable response to the current pandemic and future public health emergencies. There is, however, ongoing concern regarding access to COVID-19 vaccines in low-income countries [109]. Moreover, there is shortage of essential supplies, including oxygen, which has had a major impact on the prevention and control of the pandemic. It is, therefore, vital to transform (through good governance and financing mechanisms) the ACT-A platform to deliver vaccines, therapeutics, diagnostics, and other essential supplies [109, 110]. The global health community has the responsibility to address these inequalities so that we can collectively end the pandemic [107].

The Omicron variant has a huge role in the current wave around the world despite high vaccine coverage [111]. Omicron appears to spread rapidly around the world ever since it was identified in November 2021 [112]. It becomes obvious that vaccination alone is inadequate for controlling the infection. This has changed our understanding of the COVID-19 pandemic endgame. The emergence of new variants of concern and their spread around the world has highlighted the importance of combination prevention, including high vaccination coverage in combination with other public health prevention measures [112].

Overall, the COVID-19 pandemic and the response to it emphasise valuable lessons towards an effective and sustainable response to public health emergencies. We argue that the PHC approach captures the different preparedness and response strategies required towards ensuring health security and UHC [113]. The PHC approach enables countries to progressively realize universal access to good-quality health services (including essential public health functions) and equity, empower people and communities, strengthen multi-sectoral policy and action for health, and enhance good governance [114]. These are essential in the prevention and control of public health emergencies, to suppress transmission, and reduce morbidity and mortality [115]. Access to high-quality primary care is at the foundation of any strong health system [116], which will, in turn, have effect on containing the epidemic, and reducing mortality and CFR [117]. Australia is a good example in this regard because it has implemented a comprehensive PHC approach in combination with border restrictions to ensure health system capacity is not exceeded [56]. The PHC approach will enable countries to develop and implement a context-specific health strategy, enhance governance, strengthen their (public) health systems, minimize segmentation and fragmentation, and tackle upstream structural issues, including discrimination and socio-economic inequities [118]. This is the type of public health approach (comprehensive, equity-focused and participatory) that will be effective and sustainable to tackle public health emergencies in the twenty-first century [119, 120]. In addition, it is vital to transform the global and regional health systems, with a strong IHR and an empowered WHO at the apex [121]. We contend that this is the way towards a healthier and safer country, region and world.

## Conclusion

The COVID-19 pandemic demonstrates that the world remains vulnerable to public health emergencies with significant health and other socio-economic impacts. The pandemic takes variable shapes and forms across regions and countries around the world. The pandemic has impacted countries with inadequate governance of the epidemic, fragmentation of their health systems and higher socio-economic inequities more than others. We argue that adequate response to public health emergencies requires that countries develop and implement a context-specific national strategy, enhance governance of public health emergency, build the capacity of their health systems, minimize fragmentation, and tackle socio-economic inequities. This is possible through a PHC approach that provides universal access to good-quality health services through empowered communities and multi-sectoral policy and action for health development. The pandemic has affected every corner of the world; it has demonstrated that “no country is safe unless other countries are safe”. This should be a call for a strong global health system based on the values of justice and capabilities for health.

## Data Availability

Data are available in a public, open access repository: Johns Hopkins University: https://coronavirus.jhu.edu/data/new-cases, and UNDP: http://hdr.undp.org/en/2019-report; WHO: https://www.who.int/publications/m/item/weekly-epidemiological-update%2D%2D-22-december-2020

## Abbreviations

COVID-19: Coronavirus Disease 2019
CFRs: Case-fatality rates
HDI: Human development index
UHCI: Universal health coverage index
GHSI: Global Health Security index
HICs: High-income countries
MICs: Middle-income countries
